# A proposal for a new clinical classification of chronic pancreatitis

**DOI:** 10.1186/1471-230X-9-93

**Published:** 2009-12-14

**Authors:** Markus W Büchler, Marc E Martignoni, Helmut Friess, Peter Malfertheiner

**Affiliations:** 1 Department of General Surgery, University of Heidelberg, Germany; 2 Department of Surgery, Technical University Munich, Germany; 3 Department of Gastroenterology and Infectious Diseases, Otto von Guericke University, Magdeburg, Germany

## Abstract

**Background:**

The clinical course of chronic pancreatitis is still unpredictable, which relates to the lack of the availability of a clinical classification. Therefore, patient populations cannot be compared, the course and the outcome of the disease remain undetermined in the individual patient, and treatment is not standardized.

**Aim:**

To establish a clinical classification for chronic pancreatitis which is user friendly, transparent, relevant, prognosis- as well as treatment-related and offers a frame for future disease evaluation.

**Methods:**

Diagnostic requirements will include one clinical criterion, in combination with well defined imaging or functional abnormalities.

**Results:**

A classification system consisting of three stages (A, B and C) is presented, which fulfils the above-mentioned criteria. Clinical criteria are: pain, recurrent attacks of pancreatitis, complications of chronic pancreatitis (e.g. bile duct stenosis), steatorrhea, and diabetes mellitus. Imaging criteria consist of ductal or parenchymal changes observed by ultrasonography, ERCP, CT, MRI, and/or endosonography.

**Conclusion:**

A new classification of chronic pancreatitis, based on combination of clinical signs, morphology and function, is presented. It is easy to handle and an instrument to study and to compare the natural course, the prognosis and treatment of patients with chronic pancreatitis.

## Introduction

Chronic pancreatitis is a heterogeneous disorder with a clinical spectrum that encompasses pain, loss of exocrine pancreatic function, diabetes mellitus and various complications usually involving organs adjacent to the pancreas[1,2]). The disease may present clinically either with an individual symptom or a combination of symptoms associated with loss of pancreatic function. The single most frequent symptom of chronic pancreatitis is pain, either in the form of intermittent episodes or in a more chronic or persistent pattern[3-5].

The natural history of chronic pancreatitis is usually characterized by progression of tissue damage and various degrees of exocrine and endocrine pancreatic insufficiency, which will become apparent over time[4,6,7]. Various well-defined complications may occur at any stage during the course of the disease. However, independent of the underlying etiology, chronic pancreatitis evolves toward the same end stage, i.e., pancreatic fibrosis[8,9].

It is general knowledge that the terminal stage of chronic pancreatitis results in a loss of organ function, with the clinical manifestations of maldigestion and diabetes mellitus[10]. However, there is little knowledge of the early structural and functional abnormalities because the current diagnostic imaging procedures are not sensitive enough to visualize them and histology is normally not available in these early stages[11-13].

The presentation of very distinct clinical symptoms and the frequent complexity of their associations require a well-differentiated therapeutic approach. Current therapies in the management of chronic pancreatitis include conservative measures (analgesics, anti-inflammatory agents, enzyme replacement), endoscopic interventions as well as surgical procedures[14-18]. However, there are no guidelines for the treatment of various alterations of chronic pancreatitis, and therefore therapy is generally individualized, depending on the personal experience of the treating clinician and local logistics. Evidence-based recommendations for the treatment of chronic pancreatitis are currently lacking, but need to be implemented in the future to address the key question of whether the natural course of chronic pancreatitis can be positively influenced by different interventions, including surgery.

There is considerable controversy, for example, over the management of pain in chronic pancreatitis, with some clinicians preferring long-term conservative management, others advocating interventional endoscopies, and still others making a plea for surgical procedures[6,14,19,20]. More than half a century after the first and classical description of chronic pancreatitis by Comfort and coworkers, we are still searching for an instrument which will allow us to test and compare different therapeutic options for this disease[21].

### Attempts to classify chronic pancreatitis

The main reason for the lack of guided strategies in the therapeutic management of chronic pancreatitis is the absence of a clinically applicable classification of chronic pancreatitis. In the past, several classifications have certainly contributed to a better understanding of the pathogenesis and pathophysiology of chronic pancreatitis. The meetings in Marseilles 1963 and 1984 and in Rome 1985 added a great deal of information to our knowledge of the pathogenesis and evolution of chronic pancreatitis[22-24]. However, other than the designation of chronic alcoholic pancreatitis as a special entity of chronic obstructive pancreatitis of various etiologies, no further attempt to design a clinically relevant system of staging was undertaken[23]. In another meeting, in Cambridge 1984, the participants focused on using the emerging imaging procedure of that time, ERCP, to stage the changes in chronic pancreatitis, but again there was no staging of the clinical manifestations and no attempt was made to correlate clinical aspects with imaging[25,26].

In recent years it has become quite evident that clinical decisions cannot be based on the type and the degree of morphological abnormalities, but need to be based on clinical findings (pain, complications, pseudocysts, etc.) in combination with findings in the functional, diagnostic and imaging procedures[27].

The concept of selecting an endoscopic or a surgical procedure based primarily on the type of morphological complications has contributed significantly to the current controversy over management of pain in chronic pancreatitis[28,29].

Amman et al., organizing a symposium 1996, attempted to introduce clinical aspects into a classification of chronic pancreatitis, with special consideration of acute and chronic alcoholic pancreatitis[30]. The main result of that meeting was a better insight into different evolutional patterns of alcoholic pancreatitis, but again it was limited to alcoholic etiology and did not attempt to classify the disease in different clinical stages. Ramesh published 2002 a classification which was based on a ABC Classification system too, but included too many and redundant sub-classification parameters and therefore the classification did not gained widespread use[31]. The Manchester classification which was presented in 2006, used the ABC grading system as well but had difficulties in resembling the natural course of chronic pancreatitis and therefore needs to be further evaluated[32]. The M-ANNHEIM classification presented by Schneider et al. in 2007 tried to describe chronic pancreatitis very detailed and therefore it might be difficult to use in clinical practice[33].

### The need for a new clinical classification of chronic pancreatitis

In order to combine clinical experience in the field of chronic pancreatitis with progress in diagnostic methods and new molecular technologies for the assessment of chronic pancreatitis, a classification of chronic pancreatitis based on key clinical aspects is crucial[34-36]. This classification would not only allow us to develop a common language for the description of this disease, but also to study the dynamics of disease evolution and to compare the role of different etiologies in the appearance of chronic pancreatitis.

Such a classification needs to be applicable and transparent in all parts of the world, and would allow for comparison of experiences and serve as a basis for disease staging in clinical trials in chronic pancreatitis. A new classification should first be validated to determine whether it is suitable to be applied to the majority of patients with chronic pancreatitis, and then the value of such a classification needs to be tested in our understanding of the natural course in different etiologies (progression, arrest, regression) and most importantly, to study the clinical outcome when different therapeutic strategies are applied.

In suggesting a new classification of CP, we propose a definition of chronic pancreatitis and disease staging based on key clinical features in association with various findings obtained in current imaging techniques. Our concept for a new classification will include a rationale adopted from a liver-pancreas analogy, and will take into account established knowledge as well as the differing treatment approaches commonly used today.

### Liver-pancreas analogy in fibrogenesis

From a standpoint of morphology/histology, chronic pancreatitis evolves in a similar manner as chronic hepatitis, i.e. liver fibrosis/liver cirrhosis. In both diseases, independent of the etiology, the chronic inflammatory process results in a final morphologic picture of fibrosis/cirrhosis[37,38]. Fibrosis/cirrhosis leads to increasing functional impairment over time and finally to complete functional loss in both organs. Also, the progression from fibrosis/cirrhosis to cancer has been described in both conditions[39,40]. However, the clinical picture differs inasmuch as chronic pancreatitis is dominated by pain in addition to exocrine and endocrine functional loss.

As a hypothesis we suggest that chronic pancreatitis begins with episodes of acute inflammation with or without clinical appearance, analagous to the acute, often also clinically in apparent evolution of hepatitis from an acute into a chronic disease state (i.e., hepatitis C).

Recently, almost identical mechanisms have been described in the pathogenetic evolution of pancreatic and hepatic fibrogenesis, with a central role assigned to stellate cells for remodeling and repair of the inflammatory damage[41-44]. For staging of chronic liver disease, i.e., liver cirrhosis, the Child-Pugh classification [30], based on functional impairment and complications, has received world-wide validation and acceptance[45]. However, such a grading system has not yet been proposed in chronic pancreatitis and/or end-stage pancreatic fibrosis.

### Facts and state-of-the-art knowledge in chronic pancreatitis

1) Different etiologies lead to chronic pancreatitis with the end result of pancreatic fibrosis.

2) There are no serologic or blood markers available to diagnose/stage (grade) the disease.

3) Pathognomonic lesions of the ductular system and parenchyma are detectable by imaging.

4) Loss of exocrine and endocrine function develops during progression of the disease.

5) The end stage is characterized by steatorrhea and insulin-dependent diabetes mellitus.

6) Several characteristic complications of chronic pancreatitis are known such as common bile duct, duodenal, main pancreatic duct and vascular obstruction/stenosis.

7) Chronic pancreatitis represents a risk factor for pancreatic cancer.

8) Overall life expectancy is reduced.

### Unresolved issues in chronic pancreatitis

1) The relationship between acute pancreatitis and chronic pancreatitis is not completely defined.

2) The issues whether in chronic pancreatitis disease progression, arrest and regression of functional and morphologic findings occur is debated.

3) Diagnosis of early chronic pancreatitis by imaging is not established.

4) The role and validity of exocrine pancreatic function tests in the diagnosis is not established.

5) The pathogenesis of pain is at least multifactorial and not defined.

6) The burn-out hypothesis is still debated and not defined with regard to time evolution in different etiologies.

7) There is disagreement over whether to use enzyme treatment to influence pain.

8) The role of endoscopic intervention is not defined under evidence-based criteria.

9) The role of surgery is not defined under evidence-based criteria.

## Proposal for a clinically-based classification of chronic pancreatitis

### Definition of chronic pancreatitis (diagnosis)

For the diagnosis of chronic pancreatitis we require at least one clinical criterion (Table 1) such as pain, (recurrent) attacks of acute pancreatitis, steatorrhea, diabetes mellitus or well-defined complications (Table 2) of chronic pancreatitis. These clinical criteria must be accompanied by well-defined abnormalities in imaging findings (Table 3) or in a direct pancreatic function test.

**Table 1:** Clinical criteria

Clinical Criteria
▪pain
▪attacks of acute pancreatitis
▪complications of CP (see Table 2)
▪steatorrhea
▪diabetis mellitus

**Table 2:** Definition of complications

Definition of complications
bile duct obstruction/stenosis with cholestasis or jaundice
▪duodenal obstruction/stenosis with clinical signs
▪vascular obstruction/stenosis with clinical or morphological signs of portal/splenic vein hypertension
▪pancreatic pseudocysts with clinical signs (compression of adjacent organs, infection, bleeding, etc.)
▪pancreatic fistula (internal or external)
▪pancreatogenic ascites
▪other rare complications related to organs in vicinity (i.e., colonic stenosis, splenic pseudocyst, etc.)

**Table 3:** Imaging criteria for chronic pancreatitis

Imaging criteria for chronic pancreatitis	
**Ductal changes:**	Irregularity of the main pancreatic duct or side branches ± intraductal filling defects, calculi, duct obstruction (stricture), duct dilatation (>3 mm)
**Parenchymal changes:**	General or focal enlargement of the gland, cysts, calcifications, heterogenous reflectivity.

The etiology of chronic pancreatitis needs to be specified according to Table 4.

**Table 4:** Etiology of chronic pancreatitis

Etiology of chronic pancreatitis
▪alcohol
▪idiopathic (unknown origin)
▪hereditary
▪autoimmune or in combination with specific diseases (Crohn's, PBC)
▪tropical
▪cystic fibrosis
▪obstructive (pancreatic duct)
▪drugs

### Staging/classification of chronic pancreatitis (Stages A, B, C)

#### Specific definition of chronic pancreatitis stage A

Stage A is the early stage of chronic pancreatitis where complications have not yet appeared and the clinical exocrine and endocrine function is preserved. Subclinical signs (impaired glucose tolerance, reduced exocrine function but without steatorrhea) might already be apparent.

Stage A is accepted under the following conditions:

Pain of any type and degree and/or attacks of acute pancreatitis, no complications (Table 2), no steatorrhea, no insulin-dependent diabetes mellitus.

#### Specific definition of chronic pancreatitis stage B

Stage B is the intermediate stage where chronic pancreatitis has led to complications but clinical exocrine and endocrine function is still preserved.

The type of complication is specified (e.g., stage B, bile duct)

Stage B is accepted under the following conditions:

Patients with complications (Table 2) but without steatorrhea or diabetes mellitus.

#### Specific definition of chronic pancreatitis stage C

Stage C is the end stage of chronic pancreatitis, where pancreatic fibrosis has led to clinical exocrine and/or endocrine pancreatic function loss (steatorrhea and/or diabetes mellitus). Complications of chronic pancreatitis might or might not be present.

The type of exocrine and/or endocrine pancreatic function loss is specified (e.g., stage C, steatorrhea).

Stage C can be sub classified into three categories:

C1: Patients with endocrine function impairment

C2: Patients with exocrine function impairment

C3: Patients with exocrine/endocrine function impairment and/or complications as they are defined in table 2.

Stage C is accepted under the following conditions:

Patients with clinical manifestation of end-stage functional impairment with or without complications.

### Classification arrangement

The diagnosis of chronic pancreatitis is defined by the requested criteria (Table 1 + 3 ). The diagnosis is supplemented by the etiology (Table 4). This determination is then supplemented by the staging, potential complications (e.g., bile duct) and potential function loss (e.g., steatorrhea).

For example:

chronic pancreatitis (alcohol), stage A

chronic pancreatitis (ideopathic), stage B, bile duct

chronic pancreatitis (tropical), stage C

## Clinical evaluation of the classification

To get a first impression of the clinical practicability of the new classification we have evaluated 191 patients who were operated at the Department of General Surgery, University of Heidelberg and followed up for a period up to 3 years. The study was performed in line with the guidelines of the Declaration of Helsinki, and each patient was asked to give written informed consent for data collection as well as for publication of data in an anonymous manner.

**Figure 1:**
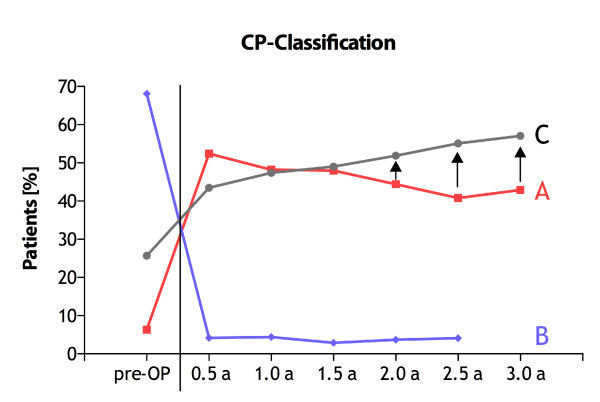
**Clinical course of chronic pancreatitis according to the classification**.

The median age of the patients was 49 years (40a/58a) (25th/75^th ^quarter). The demographic details of the patients are summarized in table 5. The type of operations are given in table 6. Patients were followed-up and restaged according to the new classification every 6 months. The classification could be applied to all patients and was easy to use. The majority of the patients were operated because of a complication (Grade B) of chronic pancreatitis (68.1%), whereas 25.7% of the patients had pancreatic insufficiency (Grade C). Interestingly, only 12 patients (6.3%) had as the only indication for operation pain, which could not be handled by medical therapy (Grade A). This finding is supported by a recent study which demonstrated a comparable incidence of complications leading to surgery[46]. A quite stable rate of 3-4.5% of the patients developed additional complications within the 6 months follow-up intervals. With a longer duration of the disease more patients developed a pancreatic insufficiency - 25.7% preoperative to 57.1% after 3a. (Table 7; Figure 1)

**Table 5:** Patients with chronic pancreatitis who were operated at the Department of General Surgery, University of Heidelberg

N = 191	N	[%]
Gender		
male	135	70.7
female	56	29.3
Etiology		
alcohol	55	33.7
idiopathic	48	29.4
biliary	16	9.8
other	44	27.0
Mortality		
alive	184	96.3
mortality (other disease)	5	2.6
mortality (reason CP)	2	1.0

**Table 6:** Indication and type of operation of patients with chronic pancreatitis who were operated at the Department of General Surgery, University of Heidelberg

N = 191	N	[%]
Indication for operation		
pain	110	57.5
stenosis of Choledochus	22	11.5
inflam. Tumor	54	28.3
pseudocyst	26	13.6
duodenal Stenosis	5	2.6
rez. Pankreatitis	26	13.6
stenosis of pancreatic duct	23	12.0
other complications	15	7.9
		
Type of operation		
duodenum preserving pancreatic head resection	86	45.0
classical Whipple	12	6.3
pp-Whipple	47	24.6
segmental resection	11	5.8
pancreatic left resection	11	5.8
pancreatico-jejunostomy	10	5.2
bili-digestive anastomosis	4	2.1
other	10	5.2

**Table 7:** Classification of patients with chronic pancreatitis throughout the follow-up period

	[pre-OP]		[0.5a]		[1a]		[1.5a]		[2a]		[2.5a]		[3a]	
	N	%	N	%	N	%	N	%	N	%	N	%	N	%
A	12	6,3%	100	52,4%	66	48,2%	49	48,0%	36	44,4%	20	40,8%	9	42,9%
B	130	68,0%	8	4,1%	6	4,4%	3	2,9%	3	3,7%	2	4,1%		
C	49	25,7%	83	43,5%	65	47,4%	50	49,1%	42	51,9%	27	55,1%	12	57,1%
	191	100,0%	191	100,0%	137	100,0%	102	100,0%	81	100,0%	49	100,0%	21	100,0%

## Discussion

The present article proposes a new clinical classification of chronic pancreatitis, which is practically easy to use and applicable for all etiologies of chronic pancreatitis.

The key concern in the management of CP arises from the inability to compare CP patients with different etiologies on different continents and from the absence of a staging/disease evolution system of chronic pancreatitis. For decades debate has continued, centering on the optimal therapeutic options. Conservative, endoscopic and surgical treatment concepts compete without being able to provide evidence of their validity at any time in the course of the disease[19,47-49].

Patients with CP are treated according to local expertise and bias, and not based on evidence-based studies. Therefore, a new classification for the definition and staging of CP is urgently needed and should serve as a basis to learn about and better understand the natural course of the disease and the effects of different interventions.

As an example of the current confusion, depending upon the different interpretations of pain mechanisms in CP, several authors advocate endoscopic interventions, i.e., stenting of the common bile duct or the main pancreatic duct or extracorporeal shock wave lithotripsy (ESWL) of pancreatic stones, whereas others deny this approach[12,28,50-52]. Furthermore, in the surgical world the debate continues about drainage operations[35-37] versus resection [17,38-41] in the management of pain and complications of CP[20,29,53-58]. In the interest of the patient, we need to carefully select, analyze and compare these measures. However, in order for a new classification to be applied, it needs to be applicable in centers around the world, and reliable assessment and comparison of the patients should be possible.

The new classification, which we propose, seems to fulfill these requirements. It is simple, reproducible and based upon clinical signs and modern imaging techniques as well as pancreatic function, and most importantly, it is also based upon our current still limited knowledge about the natural course of CP. We have taken into account the liver cirrhosis classification (Child-Pugh) as a successful model, an analogy that seems applicable in CP as well, because most recently, several authors have described common mechanisms for liver and pancreatic fibrogenesis with a resulting loss in organ function at the end stage of both diseases[41-43,59,60].

The future task should be to establish an international working group and to prospectively evaluate the clinical significance of the proposed classification. This procedure should enable us both to validate this classification and to study the course of CP with and without treatment aspects.

## Competing interests

The authors declare that they have no competing interests.

## Authors' contributions

MWB and PM carried out the idea of the classification and drafted the manuscript. HF and MEM contributed to the final version of the classification and carried out the clinical study. All authors read and approved the final manuscript.

## Pre-publication history

The pre-publication history for this paper can be accessed here:

http://www.biomedcentral.com/1471-230X/9/93/prepub

## References

[B1] DiMagno MJ Dimagno EP Chronic pancreatitis Curr Opin Gastroenterol 2006 22 487 97 16891879 10.1097/01.mog.0000239862.96833.89

[B2] Layer P DiMagno EP Early and late onset in idiopathic and alcoholic chronic pancreatitis. Different clinical courses Surg Clin North Am 1999 79 847 60 10.1016/S0039-6109(05)70047-5 10470331

[B3] Ammann RW Pain profile in alcoholic and nonalcoholic chronic pancreatitis (CP) Pancreas 1996 12 315 8 10.1097/00006676-199604000-00018 8830342

[B4] Ammann RW Muellhaupt B The natural history of pain in alcoholic chronic pancreatitis Gastroenterology 1999 116 1132 40 10.1016/S0016-5085(99)70016-8 10220505

[B5] Malfertheiner P Mayer D Büchler MW Treatment of pain in chronic pancreatitis by inhibition of pancreatic secretion with octreotide Gut 1995 36 450 4 10.1136/gut.36.3.450 7698708 PMC1382464

[B6] DiMagno EP Toward understanding (and management) of painful chronic pancreatitis Gastroenterology 1999 116 1252 7 10.1016/S0016-5085(99)70031-4 10220520

[B7] Pezzilli R Bini L Fantini L Quality of life in chronic pancreatitis World J Gastroenterol 2006 12 6249 51 17072944 10.3748/wjg.v12.i39.6249 PMC4088129

[B8] Shrikhande SV Martignoni ME Shrikhande M Comparison of histological features and inflammatory cell reaction in alcoholic, idiopathic and tropical chronic pancreatitis Br J Surg 2003 90 1565 1572 10.1002/bjs.4353 14648737

[B9] Spicak J Poulova P Plucnarova J Pancreas divisum does not modify the natural course of chronic pancreatitis J Gastroenterol 2007 42 135 9 10.1007/s00535-006-1976-x 17351802

[B10] Toskes PP Medical management of chronic pancreatitis Scand J Gastroenterol Suppl 1995 208 74 80 10.3109/00365529509107766 7777809

[B11] Manes G Kahl S Glasbrenner B Chronic pancreatitis: diagnosis and staging Ann Ital Chir 2000 71 23 32 10829520

[B12] Toskes PP Update on diagnosis and management of chronic pancreatitis Curr Gastroenterol Rep 1999 1 145 53 10.1007/s11894-996-0014-8 10980942

[B13] Nichols MT Russ PD Chen YK Pancreatic imaging: current and emerging technologies Pancreas 2006 33 211 20 10.1097/01.mpa.0000227912.71202.2c 17003640

[B14] Dumonceau JM Vonlaufen A Pancreatic endoscopic retrograde cholangiopancreatography (ERCP) Endoscopy 2007 39 124 30 10.1055/s-2006-945096 17327971

[B15] Dominguez-Munoz JE Pancreatic enzyme therapy for pancreatic exocrine insufficiency Curr Gastroenterol Rep 2007 9 116 22 10.1007/s11894-007-0005-4 17418056

[B16] van Esch AA Wilder-Smith OH Jansen JB Pharmacological management of pain in chronic pancreatitis Dig Liver Dis 2006 38 518 26 10.1016/j.dld.2006.02.002 16627019

[B17] Alexakis N Halloran C Raraty M Current standards of surgery for pancreatic cancer Br J Surg 2004 91 1410 27 10.1002/bjs.4794 15499648

[B18] Andren-Sandberg A Ansorge C Eiriksson K Treatment of pancreatic pseudocysts Scand J Surg 2005 94 165 75 16111100 10.1177/145749690509400214

[B19] Lamme B Boermeester MA Straatsburg IH Early versus late surgical drainage for obstructive pancreatitis in an experimental model Br J Surg 2007 17335122 10.1002/bjs.5722

[B20] Beger HG Schlosser W Friess HM Duodenum-preserving head resection in chronic pancreatitis changes the natural course of the disease: a single-center 26-year experience Ann Surg 1999 230 512 9 discussion 519-23 10.1097/00000658-199910000-00007 10522721 PMC1420900

[B21] Comfort M Gambill E Baggenstoss A Chronic relapsing pancreatitis Gastroenterology 1946 6 46 20985712

[B22] Sarles H Pancreatitis Symposium 1965

[B23] Singer MV Gyr K Sarles H Revised classification of pancreatitis. Report of the Second International Symposium on the Classification of Pancreatitis in Marseille, France, March 28-30, 1984 Gastroenterology 1985 89 683 5 4018507

[B24] Singer MV Chari ST Beger HG, Warshaw AL, Büchler MW, et al Classification of chronic pancreatitis The Pancreas 1998 Oxford: Blackwell Science

[B25] Axon AT Classen M Cotton PB Pancreatography in chronic pancreatitis: international definitions Gut 1984 25 1107 12 10.1136/gut.25.10.1107 6479687 PMC1432537

[B26] Axon AT Endoscopic retrograde cholangiopancreatography in chronic pancreatitis. Cambridge classification Radiol Clin North Am 1989 27 39 50 2642274

[B27] Malfertheiner P Büchler MW Correlation of imaging and function in chronic pancreatitis Radiol Clin North Am 1989 27 51 64 2642276

[B28] Dumonceau JM Deviere J Le Moine O Endoscopic pancreatic drainage in chronic pancreatitis associated with ductal stones: long-term results Gastrointest Endosc 1996 43 547 55 10.1016/S0016-5107(96)70189-X 8781931

[B29] Büchler MW Friess H Bittner R Duodenum-preserving pancreatic head resection: Long-term results J Gastrointest Surg 1997 1 13 9 10.1007/s11605-006-0004-z 9834325

[B30] Ammann RW A clinically based classification system for alcoholic chronic pancreatitis: summary of an international workshop on chronic pancreatitis Pancreas 1997 14 215 21 10.1097/00006676-199704000-00001 9094150

[B31] Ramesh H Proposal for a new grading system for chronic pancreatitis: the ABC system J Clin Gastroenterol 2002 35 67 70 10.1097/00004836-200207000-00014 12080229

[B32] Bagul A Siriwardena AK Evaluation of the Manchester classification system for chronic pancreatitis Jop 2006 7 390 6 16832136

[B33] Schneider A Lohr JM Singer MV The M-ANNHEIM classification of chronic pancreatitis: introduction of a unifying classification system based on a review of previous classifications of the disease J Gastroenterol 2007 42 101 19 10.1007/s00535-006-1945-4 17351799

[B34] Whitcomb DC Gorry MC Preston RA Hereditary pancreatitis is caused by a mutation in the cationic trypsinogen gene Nat Genet 1996 14 141 5 10.1038/ng1096-141 8841182

[B35] Friess H Yamanaka Y Büchler MW A subgroup of patients with chronic pancreatitis overexpress the c-erb B-2 protooncogene Ann Surg 1994 220 183 92 10.1097/00000658-199408000-00010 7519839 PMC1234358

[B36] Shrikhande SV Friess H di Mola FF NK-1 receptor gene expression is related to pain in chronic pancreatitis Pain 2001 91 209 17 10.1016/S0304-3959(00)00436-X 11275376

[B37] Klöppel G Maillet B Beger HG, Warshaw AL, Büchler MW, et al Pathology of chronic pancreatitis The Pancreas 1998 Oxford: Blackwell Science

[B38] Kapoor D Kumar N Hepatic fibrosis: pathobiology and management Trop Gastroenterol 2000 21 114 7 11084830

[B39] Lowenfels AB Maisonneuve P Cavallini G Pancreatitis and the risk of pancreatic cancer. International Pancreatitis Study Group N Engl J Med 1993 328 1433 7 10.1056/NEJM199305203282001 8479461

[B40] Colombo M de Franchis R Del E Ninno Hepatocellular carcinoma in Italian patients with cirrhosis N Engl J Med 1991 325 675 80 1651452 10.1056/NEJM199109053251002

[B41] Bachem MG Schneider E Gross H Identification, culture, and characterization of pancreatic stellate cells in rats and humans Gastroenterology 1998 115 421 32 10.1016/S0016-5085(98)70209-4 9679048

[B42] Schmid-Kotsas A Gross HJ Menke A Lipopolysaccharide-activated macrophages stimulate the synthesis of collagen type I and C-fibronectin in cultured pancreatic stellate cells Am J Pathol 1999 155 1749 58 10550331 10.1016/S0002-9440(10)65490-9 PMC1866993

[B43] Apte MV Haber PS Darby SJ Pancreatic stellate cells are activated by proinflammatory cytokines: implications for pancreatic fibrogenesis Gut 1999 44 534 41 10075961 10.1136/gut.44.4.534 PMC1727467

[B44] Omary MB Lugea A Lowe AW The pancreatic stellate cell: a star on the rise in pancreatic diseases J Clin Invest 2007 117 50 9 10.1172/JCI30082 17200706 PMC1716214

[B45] Child CG Turcotte JG Surgery and portal hypertension Major Probl Clin Surg 1964 1 1 85 4950264

[B46] Keck T Marjanovic G Fernandez-del C Castillo The inflammatory pancreatic head mass: significant differences in the anatomic pathology of German and American patients with chronic pancreatitis determine very different surgical strategies Ann Surg 2009 249 105 10 10.1097/SLA.0b013e31818ef078 19106684

[B47] Gupta V Toskes PP Diagnosis and management of chronic pancreatitis Postgrad Med J 2005 81 491 7 10.1136/pgmj.2003.009761 16085738 PMC1743323

[B48] Cunha JE Penteado S Jukemura J Surgical and interventional treatment of chronic pancreatitis Pancreatology 2004 4 540 50 10.1159/000081560 15486450

[B49] Weber A Schneider J Neu B Endoscopic stent therapy for patients with chronic pancreatitis: results from a prospective follow-up study Pancreas 2007 34 287 94 10.1097/mpa.0b013e3180325ba6 17414050

[B50] Binmoeller KF Jue P Seifert H Endoscopic pancreatic stent drainage in chronic pancreatitis and a dominant stricture: long-term results Endoscopy 1995 27 638 44 10.1055/s-2007-1005780 8903975

[B51] Sauerbruch T Holl J Sackmann M Extracorporeal lithotripsy of pancreatic stones in patients with chronic pancreatitis and pain: a prospective follow up study Gut 1992 33 969 72 10.1136/gut.33.7.969 1644340 PMC1379415

[B52] DiMagno EP Conservative Management of chronic pancreatitis Dig Surg 1994 11 300 303 10.1159/000172270

[B53] Nealon WH Thompson JC Progressive loss of pancreatic function in chronic pancreatitis is delayed by main pancreatic duct decompression. A longitudinal prospective analysis of the modified puestow procedure Ann Surg 1993 217 458 66 discussion 466-8 10.1097/00000658-199305010-00005 8489308 PMC1242821

[B54] Nealon WH Matin S Analysis of surgical success in preventing recurrent acute exacerbations in chronic pancreatitis Ann Surg 2001 233 793 800 10.1097/00000658-200106000-00009 11371738 PMC1421322

[B55] Falconi M Valerio A Caldiron E Changes in pancreatic resection for chronic pancreatitis over 28 years in a single institution Br J Surg 2000 87 428 33 10.1046/j.1365-2168.2000.01391.x 10759737

[B56] Sohn TA Campbell KA Pitt HA Quality of life and long-term survival after surgery for chronic pancreatitis J Gastrointest Surg 2000 4 355 64 discussion 364-5 10.1016/S1091-255X(00)80013-X 11058853

[B57] Ozawa F Friess H Kondo Y Duodenum-preserving pancreatic head resection (DPPHR) in chronic pancreatitis: its rationale and results J Hepatobiliary Pancreat Surg 2000 7 456 65 10.1007/s005340070015 11180871

[B58] Büchler MW Friess H Wagner M Pancreatic fistula after pancreatic head resection Br J Surg 2000 87 883 9 10.1046/j.1365-2168.2000.01465.x 10931023

[B59] Haber PS Keogh GW Apte MV Activation of pancreatic stellate cells in human and experimental pancreatic fibrosis Am J Pathol 1999 155 1087 95 10514391 10.1016/S0002-9440(10)65211-X PMC1867025

[B60] Jaster R Molecular regulation of pancreatic stellate cell function Mol Cancer 2004 3 26 10.1186/1476-4598-3-26 15469605 PMC524499

